# Inhibition of the Immunoproteasome Subunit LMP7 Ameliorates Cerebral White Matter Demyelination Possibly via TGF*β*/Smad Signaling

**DOI:** 10.1155/2021/6426225

**Published:** 2021-10-12

**Authors:** Xingyong Chen, Nannan Yao, Zejing Lin, Yinzhou Wang

**Affiliations:** ^1^Department of Neurology, Fujian Provincial Hospital, Shengli Clinical Medical College of Fujian Medical University, Fuzhou 350001, China; ^2^Fujian Academy of Medical Science, Fuzhou 350001, China

## Abstract

**Objectives:**

Chronic cerebral hypoperfusion induces white matter ischemic injury and cognitive impairment, whereas the mechanism remains unclear. Immunoproteasomes have been implicated in the pathogenesis of acute ischemia stroke and multiple sclerosis. However, the expression and role of immunoproteasomes in the brain of chronic cerebral hypoperfusion remain to be clarified.

**Methods:**

Chronic white matter ischemic injury mice models were induced by bilateral carotid artery stenosis (BCAS). A selective immunoproteasome subunit low-molecular-mass peptide-7 (LMP7) inhibitor PR957 was administered to mice. Cognitive function, white matter integrity, and potential pathways were assessed after BCAS.

**Results:**

The present study found that chronic cerebral hypoperfusion following BCAS induced cerebral white matter demyelination and cognitive impairment, accompanied with elevated expression of the immunoproteasomes LMP2 and LMP7, activation of astrocytes and microglia, and increased production of inflammatory cytokines (e.g., interleukin-1*β* (IL-1*β*), tumor necrosis factor-*α* (TNF-*α*), IL-10, transforming growth factor-*β*1 (TGF*β*1), and insulin-like growth factor-1 (IGF-1)). However, inhibition of LMP7 with the specific proteasome inhibitor PR957 significantly mitigated the histological damage of the white matter, suppressed inflammatory response, and paralleled by an improvement of cognitive function. Furthermore, treatment of PR957 significantly upregulated the level of TGF*β*1, the total expression level, and the phosphorylation level of Smad2/3 and promoted brain remyelination. Surprisingly, PR957 alone had no effects on the neuroinflammation response and the activation of TGF*β*/Smad signaling in the sham-operated (BCAS-nonoperated) mice.

**Conclusions:**

The possible mechanism underlying this was attributed to that the immunoproteasome regulates TGF*β*/Smad signaling-mediated neuroinflammation and oligodendrocyte remyelination.

## 1. Introduction

The white matter mainly consists of myelinated axons and myelin-forming cells and functions to transduct nervous impulses. Characterized by loss of axon-glial integrity and demyelination, white matter injury often occurs in various diseases such as multiple sclerosis [1], stroke [2], chronic cerebral ischemia, and even vascular dementia [3]. Growing evidence demonstrates that chronic cerebral hypoperfusion, which is commonly accompanied by cognitive impairment, can induce the excessive inflammatory responses and myelin damage linked to some extent and occurs simultaneously [4, 5]. It is assumed that demyelination is often associated with an inflammatory response manifested by cellular activation of microglia and astrocytes and production of various inflammatory cytokines [5, 6]. Demyelination and inflammation can be causative and interact with each other; a moderate inflammatory response contributes to myelin regeneration, while an excessive inflammatory response aggravates myelin destruction and eventually leads to the aggravation of pathological damage [7]. However, the underlying cause has not yet been entirely clear. Accordingly, several myelin-protective or promoting remyelination strategies are designed to attenuate the inflammatory response.

Myelination is affected by numerous intrinsic cellular mechanisms and extrinsic stimulus factors [8]. It has been confirmed that transforming growth factor-*β* (TGF*β*) signaling is an important cue for oligodendrocyte proliferation and differentiation, myelination, and remyelination [9, 10]. TGF*β* regulates gene transcription through Smad proteins and signals via non-Smad pathways. The interaction of TGF*β* with its receptors (including TGF*β*-RI and TGF*β*-RII) can induce the phosphorylation of RSMAD (receptor-regulated SMAD) and formation of complexes with the co-mediator SMAD (Co-SMAD). These complexes are then translocated to the nucleus and regulate the transcriptional activity of the target genes [11]. There is ample evidence that endogenous TGF*β* has been demonstrated to be one of the protective factors against multiple sclerosis [10]. Although the crucial role of TGF*β* signaling in central nervous system (CNS) myelination has been recognized [9, 10], the exact molecular mechanisms underlying remain unclear. On the other hand, TGF*β* signaling is tightly regulated by the ubiquitin-proteasome system (UPS), which is the major pathway of nonlysosomal proteolysis of intracellular proteins [12–14]. The UPS contributes to the regulation of Smad and Smad ubiquitination-related factor (Smurf) protein levels during Smad activation and subsequently affects TGF*β* signaling function. Therefore, approach interference with components of the UPS may affect TGF*β* signaling transduction.

Immunoproteasomes consist of three catalytic subunits: *β*1i (also termed low molecular mass peptide-2, LMP2), *β*2i (LMP5), and *β*5i (LMP7). Immunoproteasome-mediated proteolysis displays roles in immune and nonimmune functions [15], and it has been linked to a variety of central nervous system diseases such as multiple sclerosis (MS) [16], stroke [17], and Alzheimer's disease [18]. They are expressed in multiple cell types such as neurons, endothelial cells, oligodendrocytes, astrocytes, macrophages/microglia, and lymphocytes [16, 19, 20]. Moreover, the immunoproteasome subunit LMP2 60HH variant reduces the risk to develop MS amongst Italian HLAA∗02+ females [16]. Inhibition of immunoproteasomes may contribute to prevent demyelization to some extent in the central nervous system (CNS). It was reported that a selective inhibition of the immunoproteasome subunit LMP7 blocked inflammation cytokine production and attenuated the progression of disease [21, 22]. These data suggest that immunoproteasome inhibitors are promising drugs for the treatment of inflammation-associated diseases. In addition, our previous study confirmed the immunoproteasome involved in the inflammatory pathophysiological mechanisms of ischemia stroke in rats after middle cerebral artery occlusion (MCAO) [20]. Whether there is a similar phenomenon in chronic cerebral ischemia models needs further observation. In the present study, using a mouse model of white matter ischemic damage caused by chronic hypoperfusion following bilateral carotid artery stenosis (BCAS), we investigated the expression of immunoproteasome and its relationship with white matter injury as well as the possible mechanisms of immunoproteasome inhibition by PR957-exerted neuroprotective effects.

## 2. Materials and Methods

### 2.1. Ethical Approval and Experimental Animals

All animal studies were approved by the Institutional Animal Ethical Committee of Fujian Provincial Hospital and performed according to the guidelines of the US Department of Health for Use and Care of Laboratory Animals. Chronic cerebral hypoperfusion models were established by bilateral carotid artery stenosis (BCAS) as described previously [23]. Briefly, adult male C57Bl/6 mice were anesthetized with sodium pentobarbital. Through a midline cervical incision, both common carotid arteries were exposed. A microcoil (0.18 mm internal diameter; Sawane Spring Co, Shizuoka, Japan) was applied to the bilateral common carotid artery. Sham-operated animals underwent identical procedures without a microcoil placed around the arteries. Core body temperatures were maintained between 36.5°C and 37.5°C during the whole procedure. After BCAS, the cerebral blood flow (CBF) was measured by laser-Doppler flowmetry at 1, 7, and 30 days. The animals were kept in cages for 30 days with free access to food and water until sacrificed at the indicated time. Mice were randomly assigned into 4 groups (each group *n* = 10): sham-operated mice treated with vehicle (sham group), sham-operated mice treated with PR957, BCAS mice treated with PR957 (PR957 group), and BCAS mice treated with vehicle (vehicle group).

### 2.2. Drug Administration

PR957 (also called ONX0914) is one novel inhibitor of the immunoproteasome subunit LMP7 and is characterized by high selectivity [24]. PR957 (MedChemExpress, USA) was formulated in an aqueous solution of 10% (w/v) sulfobutylether-*β*-cyclodextrin and 10 mM sodium citrate (pH 6.0) and administered to mice as an s.c. dose of 10 mg/kg (in a volume of 100 mL) as described previously [22].

### 2.3. Eight-Arm Radial Maze Test

Working memory was assessed by an eight-arm maze test conducted one month after BCAS as described previously [23, 25]. All mice were pretrained, and the test was repeated 8 times using 8 different arms. Spatial working memory and reference memory tasks were completed following the literature [23, 25] and recorded by observers who were blinded to the experiments.

### 2.4. Tissue Preparation

At 1 month after surgery, mice were euthanized with 5% isoflurane and transcardially perfused with ice-cold 50 mL of 0.01 M phosphate-buffered saline (PBS). For Western blot and enzyme-linked immunosorbent assay (ELISA), brains were removed and rapidly frozen in liquid nitrogen. For immunohistochemistry and immunofluorescence, mice were then perfused with 20 mL of 4% paraformaldehyde (PFA) in 0.01 M PBS. After perfusion, brains were removed, postfixed in 4% PFA overnight (4°C), and cryoprotected using 20%–30% sucrose in PBS. All brains were stored in −80°C until processing.

### 2.5. Luxol Fast Blue (LFB) Staining

LFB is commonly used to detect demyelination in the CNS. Coronal sections (20 *μ*m) were stained with LFB according to the manufacturer's instructions (Solarbio Life Science, Beijing, China) to detect myelin damage. In brief, the slides were incubated in LFB solution for 14 hours at 60°C, after water rinse, the sections were differentiated by dipping in lithium carbonate solution and then differentiated further by dipping in an alcohol reagent. The severity of the WM lesions was graded as normal (grade 0), disarrangement of the nerve fibers (grade 1), formation of marked vacuoles (grade 2), and the disappearance of myelinated fibers (grade 3) as described [26].

### 2.6. Enzyme-Linked Immunosorbent Assay (ELISA)

Concentrations of IL-1*β*, TNF-*α*, TGF*β*1, insulin-like growth factor-1 (IGF-1), and IL-10 proteins in brain tissues from the corpus callosum (CC), forebrain cerebral cortex, and striatum were determined by the use of commercially available ELISA kits (Shanghai Meilian Biological Technology Co., Ltd., Shanghai, China) according to the manufacturer's instructions. All samples and standards were measured in duplicate, and the average value was recorded (pg/mg).

### 2.7. Immunofluorescence (IF)

Briefly, brain slices were preincubated with 0.1% Triton X-100 (v/v) in 0.01 M PBS (pH 7.4) for 15 minutes. After blocking with 10% normal goat serum (Sigma-Aldrich), slides were incubated with primary antibodies as follows: rabbit anti-GFAP, rabbit anti-myelin basic protein (MBP), and rabbit anti-IBA1 (1 : 400; Abcam, Cambridge, MA, USA). After incubated overnight at 4°C and rinsed in 0.01 M PBS (3 × 5 minutes), tissue slices were incubated with Alexa Fluor® 488 conjugated goat anti-rabbit IgG (*H* + *L*), F(ab')2 Fragment or Alexa Fluor® 594 conjugated goat anti-mouse IgG (*H* + *L*), F(ab')2 Fragment (1 : 1000; Cell Signaling Technology) in 0.01 M PBS for 1 hour at room temperature. If necessary, the sections were counterstained for nuclei with 4′,6-diamidino-2-phenylindole dihydrochloride (DAPI; 1 : 1000; Roche, Mannheim, Germany), and then slides were mounted in the ProLong® Gold antifade reagent (Thermo Fisher Scientific, USA) prior to imaging. The immunofluorescence intensity was analyzed with Image *J*, v1.8.0.

### 2.8. Western Blot Analyses

Western blot was performed as described previously [20]. Briefly, total protein (20–40 *μ*g) from the corpus callosum, forebrain cerebral cortex, and striatum was separated by 4–20% gradient SDS/PAGE (sodium dodecyl sulfate polyacrylamide gel electrophoresis) and then transferred onto polyvinylidene fluoride (PVDF) membranes (Millipore, USA) using a Bio-Rad Trans-Blot® transblot module (Bio-Rad, Hercules, CA, USA). After blocking with Tris-buffered saline containing 0.1% Tween-20 (TBST) and 5% nonfat milk (Solarbio Life Science, Beijing, China), the membranes were then incubated with primary antibodies. Primary antibodies used were as follows: rabbit anti-LMP2, rabbit anti-LMP7, rabbit anti-TGF*β*1, rabbit anti-Smad2 (phospho T8) + Smad3 (phospho T8), rabbit anti-myelin basic protein (MBP) (1 : 1000; Abcam, Cambridge, MA, USA), and mouse monoclonal anti-*β*-actin (1 : 3000; Cell Signaling Technology, USA). Membranes were exposed to secondary antibodies diluted in blocking buffer for 1 hour at room temperature: horseradish peroxidase (HRP)-conjugated goat anti-rabbit or HRP-conjugated goat anti-mouse IgG antibody (1 : 3000; Cell Signaling Technology, USA). Immunoreactivity was detected with the chemiluminescent HRP substrate (Thermo Scientific™, USA). The optical densities were normalized to that of *β*-actin and calculated as target protein expression/*β*-actin expression ratios (Image *J*, v1.8.0).

### 2.9. Measurements of 20S Proteasome Activity

Proteasome Activity Assay Kit (Chemicon, Billerica, MA, USA) was used to detect the proteasome activity as described previously [20]. In brief, to determine chymotrypsin- and caspase-like activities, 150 *µ*g of protein was incubated in assay buffer including 250 mmol/L HEPES HCl, pH 7.5, 5 mmol/L EDTA, 0.5% Nonidet-P40, and 0.1% sodium dodecyl sulfate (SDS) (w/v), with 50 *μ*mol/L substrate (Z-Leu-Leu-Glu-AMC for caspase-like activity; Suc-Leu-Leu-Val-Tyr-AMC for chymotrypsin-like activity) in a final volume of 100 *μ*L in 96-well plates for 1 hour at 37°C according to the manufacturer's instructions. The fluorescence of each solution was monitored by detecting the release of AMC with spectrophotometry (Thermo Electron, USA) at an excitation wavelength of 380 nm and an emission wavelength of 440 nm.

### 2.10. Statistical Analysis

Parametric data from different groups were compared using one-way ANOVA followed by the least significant difference (LSD) test. All data are presented as mean ± standard deviation. A value of *P* < 0.05 was considered statistically significant.

## 3. Results

### 3.1. Inhibition of LMP7 Attenuates Chronic Cerebral Hypoperfusion-Induced White Matter Demyelination and Cognitive Impairment

**Figure 1:**
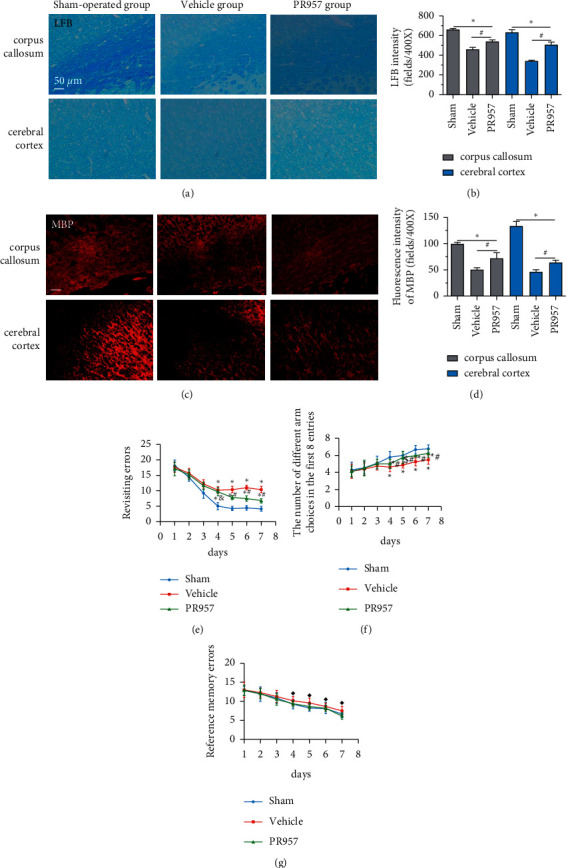
Inhibition of LMP7 attenuates chronic cerebral hypoperfusion-induced white matter demyelination and cognitive impairment. LFB staining confirmed the disruption of white matter integrity in the corpus callosum (CC) and forebrain cerebral cortex in mice 30 days after hypoperfusion (a, b). Immunofluorescence showed the expression of MBP in different groups (c, d). Working memory was assessed by an eight-arm maze test conducted one month after BCAS (e–g). Results were expressed as mean ± standard deviation from three independent experiments. ^*∗*^*P* < 0.001, ^$^*P* < 0.01, and ^◆^*P* > 0.05 compared with the sham-operated group; ^#^*P* < 0.001 and ^&^*P* > 0.05 compared with the vehicle group. Bar = 50 *μ*m. *n* = 10.

First, we evaluated the white matter demyelination lesion by LFB staining at 1 month after hypoperfusion. As shown in Figure 1, LFB staining confirmed the disruption of white matter integrity in the corpus callosum (CC) and forebrain cerebral cortex in mice 30 days after hypoperfusion (Figures 1(a) and 1(b)). Similarly, immunofluorescence (IF) showed that there were weaker immunities of MBP in BCAS-operated mice than the sham-operated mice (Figures 1(c) and 1(d)). Both LFB and IF indicated the most severe rarefaction was in the medial part of the corpus callosum. On the other hand, inhibition of the LMP7 with administration of PR957 significantly reversed the histological damage of white matter. Compared with the vehicle group, immunofluorescence intensities of LFB and MBP in the PR957 group were higher (*P* < 0.001).

Next, cognitive impairment was detected in mice with cerebral hypoperfusion. Interestingly, mice in the sham-operated group quickly improved their performance in the working memory task of the 8-arm radial maze, while BCAS-operated mice took significantly more revisiting errors and fewer different arm choices in the first 8 entries (*P* < 0.001) (Figures 1(e)–1(g)). Furthermore, compared with the vehicle group, inhibition of the LMP7 with PR957-treated improved performance in both revisiting sessions and different choice counts, suggesting an improvement in short-term memory (*P* < 0.001) (Figures 1(e)–1(g)). However, there were no significant differences among the three groups in the spatial reference memory task of the 8-arm radial maze (*P* > 0.05) (Figures 1(e)–1(g)).

### 3.2. Chronic Hypoperfusion Upregulates the Expression and Proteasome Activity of Immunoproteasome and Is Attenuated by Treatment with PR957

**Figure 2:**
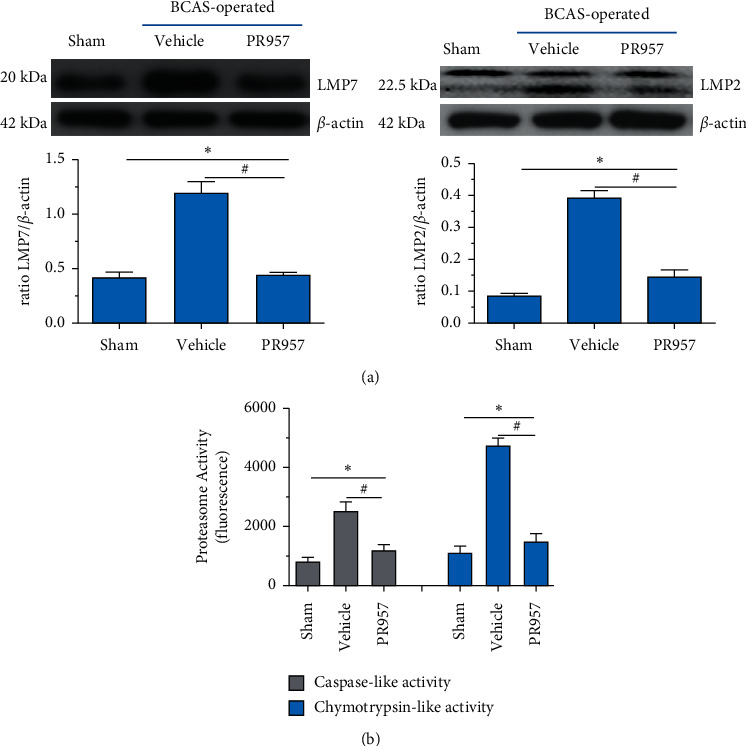
Chronic hypoperfusion upregulates the expression and proteasome activity of immunoproteasomes and is attenuated by treatment with PR957. Western blot confirmed that LMP2 and LMP7 protein levels were elevated after 1-month hypoperfusion, whereas the intensity tended to decrease in mice treated with PR957 (^*∗*^*P* < 0.001, compared with the sham-operated group; ^#^*P* < 0.001, compared with the vehicle group) (a). The chymotrypsin- and caspase-like activities in different groups (b). Results were expressed as mean ± standard deviation from three independent experiments. ^*∗*^*P* < 0.001, compared with the sham-operated group; ^#^*P* < 0.001, compared with the vehicle group, *n* = 10.

Our previous study found that immunoproteasomes involved in the mechanism of ischemia stroke and inhibition of the immunoproteasome LMP2 suppressed inflammation and enhanced angiogenesis in MCAO rats [20, 27]. In the present study, we aim to confirm whether the expressions of the immunoproteasome subunits LMP2 and LMP7 and proteasome-dependent proteolytic activities increased at 1 month after BCAS. As shown in Figure 2, compared with the sham-operated group, both LMP2 and LMP7 protein levels were elevated in the vehicle and PR957 groups after 1-month hypoperfusion. However, inhibition of the LMP7 with PR957 significantly reversed the downregulation of LMP2 and LMP7 proteins related to the vehicle groups (*P* < 0.001) (Figure 2(a)).

We further measured the proteasome-dependent proteolytic activity 30 days after hypoperfusion. Hypoperfusion induced a robust increase in chymotrypsin- and caspase-like activities (about 3-fold and 4-fold increase compared with the sham-operation mice, respectively), whereas administration of PR957 significantly caused nearly 52.3% and 68.3% decrease in caspase- and chymotrypsin-like activities compared with the vehicle group, respectively (*P* < 0.001) (Figure 2(b)).

### 3.3. Inhibition of LMP7 Attenuates Chronic Hypoperfusion-Induced Neuroinflammation Response

**Figure 3:**
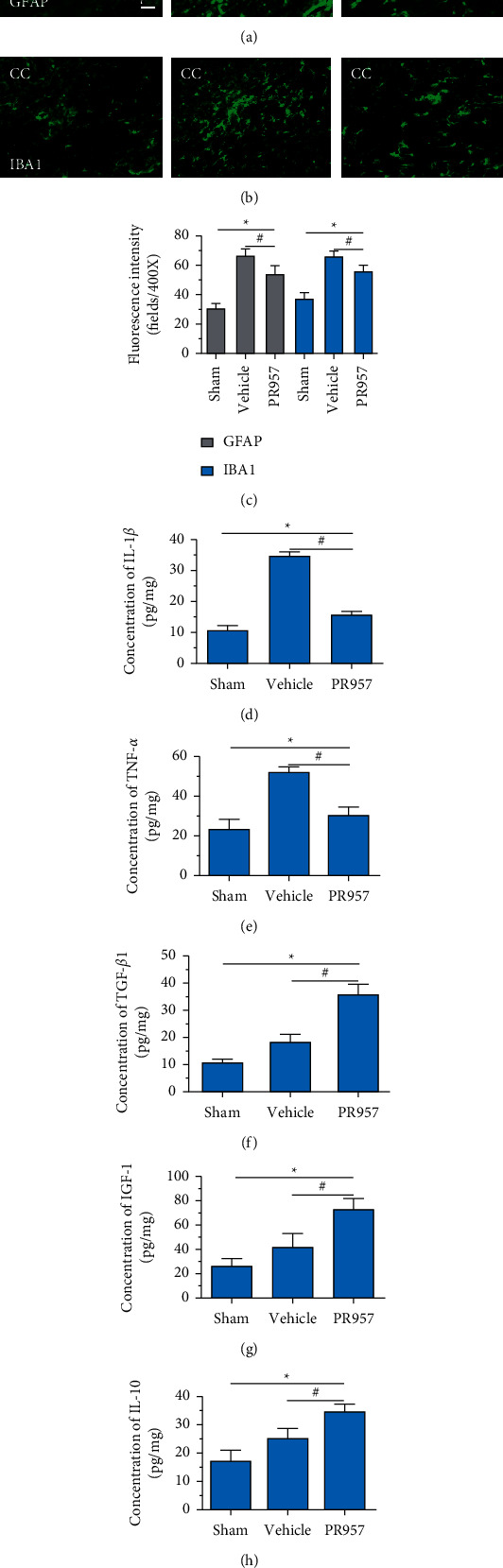
Inhibition of LMP7 attenuates chronic hypoperfusion-induced neuroinflammation response. Immunofluorescence showed that GFAP and IBA1 immunoreactivities were evident in the corpus callosum in BCAS-operated mice compared with sham-operated animals. In contrast, GFAP and IBA1 immunoreactivities were significantly reduced in mice treated with PR957 (*P* < 0.001) (a–c). ELISA tested the levels of IL-1*β*, TNF-*α*, TGF*β*1, IGF-1, and IL-10 proteins in three groups (d–h). Results were expressed as mean ± standard deviation from three independent experiments. ^*∗*^*P* < 0.001, compared with the sham-operated group; ^#^*P* < 0.001, compared with the vehicle group. Bar = 50 *μ*m. *n* = 10.

To investigate the potential links between inflammation and white matter demyelination, we examined the activation of astrocytes, microglia, and the expression of proinflammatory cytokines IL-1*β* and TNF-*α*, as well as anti-inflammatory cytokines TGF*β*1, IGF-1, and IL-10. In the present study, we found that chronic cerebral ischemia induces an enlarged inflammatory response. Following BCAS, both GFAP positive of astrocytes and IBA1 positive of microglia were activated in the brain of BCAS-operated mice. Immunofluorescence showed that there were weak GFAP and IBA1 immunoreactivities for astrocytes and microglia in the corpus callosum (CC) in sham-operated animals, respectively. Following BCAS, both GFAP and IBA1 immunoreactivities were evident in the corpus callosum in BCAS-operated mice. In contrast, GFAP and IBA1 immunoreactivities were significantly reduced in mice treated with PR957 (*P* < 0.001) (Figures 3(a)–3(c)).

Similarly, ELISA confirmed that the levels of IL-1*β*, TNF-*α*, TGF*β*1, IGF-1, and IL-10 proteins were significantly elevated in BCAS groups compared with the sham-operated group (*P* < 0.001) (Figures 3(d)–3(h)). Compared with the vehicle group, there was a significantly decreased concentration of IL-1*β* and TNF-*α* protein and increased concentration of TGF*β*1, IGF-1, and IL-10 in the PR957 group (*P* < 0.001) (Figures 3(d)–3(h)). Taken together, these data suggest that there is a correlation between neuroinflammation and white matter lesions, and inhibition of the immunoproteasome LMP7 attenuates the inflammatory response.

### 3.4. Inhibition of LMP7 Promotes the Activation of TGF*β*/Smad Signaling and Remyelination after Chronic Cerebral Hypoperfusion

**Figure 4:**
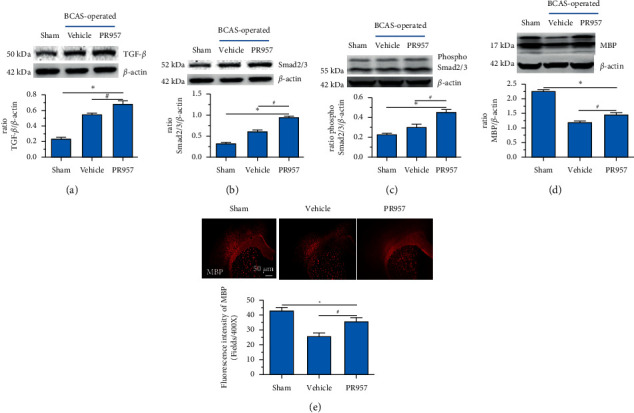
Inhibition of LMP7 promotes the activation of TGF*β*/Smad signaling and remyelination after chronic cerebral hypoperfusion. Western blot confirmed the levels of TGF*β*1, Smad2/3, and MBP protein in different groups (a–d). Immunofluorescence staining indicated the protein expression of MBP in different groups (e). Results were expressed as mean ± standard deviation from three independent experiments. ^*∗*^*P* < 0.001, compared with the sham-operated group; ^#^*P* < 0.001, compared with the vehicle group. Bar = 50 *μ*m. *n* = 10.

Previous study reported that TGF*β*/Smad signaling modulates CNS myelination [9]. In the present study, we found the activation of TGF*β*/Smad signaling at 1-month hypoperfusion after BCAS. Compared with the sham-operated group, the levels of TGF*β*1 and Smad2/3 protein were significantly increased in the BCAS group. Moreover, inhibition of the LMP7 with treatment of PR957 upregulated the level of TGF*β*1, the total protein expression level, and the phosphorylation level of Smad2/3 compared with the vehicle group (*P* < 0.001) (Figures 4(a)–4(c)). Western blot confirmed that the protein expression of TGF*β*1, the total protein, and the phosphorylation level of Smad2/3 were enhanced with PR957 treatment (*P* < 0.001) (Figures 4(a)–4(c)).

On the other hand, we found inhibition of the LMP7 promoted brain remyelination after chronic cerebral hypoperfusion. As shown in Figures 4(d) and 4(e), both Western blot and immunofluorescence staining indicated that the protein expression of MBP, one of the most important components of myelination, was markedly reduced after one-month hypoperfusion, while inhibition of the LMP7 with administration of PR957 significantly reversed the downregulated level of MBP (*P* < 0.001).

### 3.5. Effects of PR957 on the Neuroinflammation Response and the Activation of TGF*β*/Smad Signaling in the Sham-Operated Group

To demonstrate whether PR957 alone has effect on the neuroinflammation response and the activation of TGF*β*/Smad signaling in the sham-operated (BCAS-nonoperated) mice, the levels of IL-1*β*, TNF-*α*, TGF*β*1, IGF-1, and IL-10 proteins as well as the levels of TGF*β*1 and Smad2/3 proteins were detected by ELISA or Western blot, respectively. As shown in Supplemental Figure 1, no significant changes were found in IL-1*β*, TNF-*α*, TGF*β*1, IGF-1, and IL-10 protein levels between sham-operated mice treated with vehicle and sham-operated mice treated with PR957 (*P* > 0.05). Similarly, Western blot confirmed that there was no significant difference in the levels of TGF*β*1, Smad2/3, and phospho-Smad2/3 protein in sham-operated groups treated with vehicle or PR957 (Supplemental Figure 2) (*P* > 0.05). These data suggest that PR957 alone had no effects on the neuroinflammation response and the activation of TGF*β*/Smad signaling in the sham-operated mice.

## 4. Discussion

Inflammation is regarded as an important risk factor for dementia [28], stroke [29], small vessel disease [30], and multiple sclerosis [31]. Chronic cerebral hypoperfusion usually leads to a persistent proinflammatory microenvironment including recruitment of immune inflammatory cells and production of multiple inflammatory mediators. Both white matter injury and inflammation are the common histopathology characteristics of chronic cerebral hypoperfusion models [31]. Particularly, this pathological change is most pronounced in multiple sclerosis disease [1]. Therefore, neuroinflammation is considered an important underlying mechanism that causes white matter injury and myelination damage. During the process of disease, microglia/macrophages and astrocytes are the most potent modulators of CNS repair/regeneration. However, these cells appear to be double-edged swords in the battle for neurological recovery [32]. Under inflammation condition, both microglia/macrophages and astrocytes shift their polarization into different phenotypes at different stages of injury. For example, the microglia/macrophage phenotype can exert positive or negative effects on neurogenesis after injury. M1 microglia/macrophages typically release destructive proinflammatory mediators (e.g., IL-1*β*, TNF-*α*). In contrast, M2 phenotypes clear cellular debris through phagocytosis and release numerous protective/trophic factors (e.g., TGF*β*1, IGF-1, and IL-10) [32]. Similarly, reactive astrocytes also have two different phenotypes, A1 and A2, which play neurotoxic and neuroprotective roles, respectively [33, 34]. Neurotoxic A1 astrocytes secrete toxic factors (e.g., IL-1*β*, TNF-*α*, and NO) that kill mature oligodendrocytes and neurons. In comparison, the neuroprotective A2 reactive astrocytes can upregulate many neurotrophic factors (e.g., IL-4, IL-10, and TGF*β*). More interestingly, microglia can induce the transformation of A1/A2 reactive astrocytes, and they cooperatively regulate neuronal activity, synaptic transmission, and myelin regeneration [34–36]. In the present study, we found that chronic cerebral ischemia induces an enlarged inflammatory response. Following BCAS, both GFAP positive of astrocytes and IBA1 positive of microglia were activated in the brain of BCAS-operated mice. In addition, ELISA confirmed that the levels of the proinflammatory mediators IL-1*β* and TNF-*α* as well as the anti-inflammation mediators TGF*β*1, IGF-1, and IL-10 were also significantly elevated in BCAS groups compared with the sham-operated group. Moreover, inhibition of the LMP7 attenuated the inflammatory response and decreased the activation of microglia/macrophages and astrocytes and the levels of the proinflammatory mediators IL-1*β* and TNF-*α*. In contrast, inhibition of the LMP7 led to the increase of protective/trophic factors TGF*β*1, IGF-1, and IL-10. One explanation for these dualistic effects of the LMP7 inhibition on microglia/macrophages and astrocytes in our study was their polarization into different phenotypes at different stages of injury. Recent studies in various CNS injury models show that anti-inflammation or immunosuppressant treatment contributes to protect myelination damage and promote white matter repair [25, 37]. Correspondingly, we also observed that inhibition of the LMP7 mitigated histological damage of the white matter, attenuated the inflammatory response, and promoted remyelination. In addition, we surprisingly found that PR957 alone had no effects on the neuroinflammation response and the activation of TGF*β*/Smad signaling in the sham-operated (BCAS-nonoperated) mice. Thus, appropriate combating inflammation is considered to be a therapeutic strategy for the CNS injury, and the LMP7 may be one of the ideal candidate targets for inflammatory response.

However, the neuroinflammatory response is rigorously mediated by multiple complex factors and mechanisms. The UPS is the major pathway of nonlysosomal proteolysis of intracellular proteins. It plays important roles in a variety of fundamental cellular processes including regulation of cell cycle progression, apoptosis, and immune and inflammatory responses [38]. Immunoproteasomes are a subtype of proteasomes and play a critical role in regulating the production of proinflammatory cytokines and maintaining protein homeostasis [27, 39]. For example, immunoproteasomes and PA28-*αβ* regulator are present in multiple sclerosis-affected brain areas, accumulated in plaques, and involved in MS development [16]. In addition, the augmentation of immunoproteasomes involves in the inflammatory mechanisms of ischemia stroke and inhibition of the immunoproteasome subunit LMP2 offers suppression of proinflammatory cytokines after MCAO [20]. In this study, we found that the immunoproteasome was also markedly upregulated in cerebral tissues of chronic cerebral hypoperfusion after BCAS. Moreover, inhibition of the immunoproteasome LMP7 with PR957 displayed suppression of the neuroinflammation response and mitigated white matter injury and improvement of cognitive impairment. To our surprise, we found that both the levels of LMP7 and LMP2 proteins and the proteolytic activities decreased after LMP7 inhibitor treatment. The possible explanation for this was that the entirety of inflammation environment improved after PR957 treatment. To the best of our knowledge, this is the first experimental evidence to indicate a protective effect and the possible underlying mechanism of inhibition of the immunoproteasome LMP7 in hypoperfusion-induced white matter injury. Indeed, inhibition of the immunoproteasome LMP7 with PR957 (or named ONX0914) or genetic ablation of LMP7 gene (LMP7−/−) could attenuate the inflammation response in in vivo and in vitro experiments [21, 22, 40]. A recent study has shown that ONX0914 treatment prevented the disease exacerbation of experimental autoimmune encephalomyelitis [22]. Interestingly, *β*5i/LMP7-deficient mice expressed more anxiety after mild stress and increased cued fear after fear conditioning [41], suggesting LMP7 implicated in the pathophysiological mechanism of emotion behavior. Taken together, this evidence supports the hypothesis of the involvement of the immunoproteasome in the pathogenesis of chronic hypoperfusion-induced white matter injury.

Myelination by oligodendrocytes in the CNS is essential for proper brain function, but the precise molecular determinants that control this process remain unclear. Accumulated evidence confirms that TGF*β* signaling may be an important cue for oligodendrocyte proliferation and differentiation, myelination, and remyelination [8, 9, 11]. As is known to us, TGF*β* signaling plays crucial roles in the regulation of cell behaviors such as antiproliferative action and inducing differentiation on multiple cell types (e.g., astroglial and neural progenitor cells) through Smad proteins and signals via non-Smad pathways [9, 42]. Mechanistically, TGF*β* signaling in oligodendrocytes modulates the canonical downstream TGF*β*-R effectors SMAD2/3/4. Upon TGF*β*-R activation, the heteromeric SMAD2/3/4 complex establishes nuclear localization and cooperates with FoxO1 and Sp1 to modulate the transcription of c-myc and p21 [9]. Notably, we cannot exclude the possibility that TGF*β* signaling can also modulate other TFs that positively or negatively influence oligodendrocytes development during CNS myelination. On the other hand, the TGF*β* pathway is strictly regulated by the UPS [12, 13, 42]. It is important to note that the ubiquitin-mediated proteasomal degradation pathway is an evolutionary conserved cascade that tightly regulates TGF*β* superfamily signaling. Both the size of the Smad pool in unstimulated cells and Smad protein levels subsequent to the activation of the pathway are controlled by ubiquitination [12]. Recent studies reported that OTUB1 can enhance TGF*β* signaling by inhibiting the ubiquitination and degradation of the active phospho-SMAD2/3 complex, while depletion of OTUB1 in cells causes a rapid loss in the levels of TGF*β*-induced phospho-SMAD2/3, which is rescued by the proteasomal inhibitor bortezomib [43]. Therefore, these proofs confirmed that the UPS tightly regulates the TGF*β*/Smad signaling pathway. Thus, TGF*β* signaling is a promising therapeutic target for the treatment of multiple sclerosis and other autoimmune diseases because of its capacity to modulate immune cell functions [10]. Previous studies have reported that systemic administration of TGF*β*1 attenuates experimental autoimmune encephalomyelitis (EAE) in mice, suggesting a protective role of TGF*β* in EAE [10]. In the current study, we observed that chronic cerebral hypoperfusion following BCAS induced TGF*β* signaling pathway activation, indicating upregulation of TGF*β*1 and Smad2/3 proteins, which potentially exerts a protective role for remyelination. In addition, treatment with PR957 further increased the protein levels of TGF*β*1, Smad2/3, MBP, and protective cytokines such as IGF-1 and IL-10 and mitigated histological damage of the white matter and promoted remyelination. Taken together, we suggested the possible underlying mechanisms responsible for the neuroprotection of LMP7 inhibition were attributed to TGF*β*/Smad signaling-mediated modulation of a better microenvironment facilitated for myelin repair and regeneration in white matter chronic ischemic injury.

The present study has several limitations. Both activated microglia and astrocytes can shift their polarization into different phenotypes and exert different roles under injury conditions. Thus, these cells appear to be double-edged swords in the battle for neurological recovery. We did not stain the activated microglia and astrocytes by use of different antibodies (for example, IBA1 and CD68 markers for microglia/macrophages), although we found the decreased production of proinflammation cytokines IL-1*β* and TNF-*α* and the elevation of anti-inflammation cytokines TGF*β*1, IL-10, and IGF-1. In addition, there is a lack of in vitro experiments further to verify the hypothesis whether PR957 treatment protects against chronic ischemic white matter damage by modulating microglia or astrocytes polarization via the TGF*β*/Smad pathway. All in a word, further studies are required to understand the mechanisms by which inhibition of the LMP7 ameliorates cerebral white matter demyelination injury.

## 5. Conclusion

Chronic cerebral hypoperfusion following BCAS caused cerebral white matter demyelination and cognitive impairment, accompanied with elevated expression of immunoproteasomes, activation of astrocytes and microglia, and increased production of inflammatory cytokines. Inhibition of the LMP7 mitigated histological damage of the white matter and inflammation response, activation of TGF*β*/Smad signaling, and improvement of cognitive impairment. The possible mechanism underlying this was attributed to the immunoproteasome regulates TGF*β*/Smad signaling-mediated neuroinflammation and oligodendrocyte remyelination.

## Data Availability

All the data involved in this study are included along with this article. The materials are available from commercial sources. There are no security, licensing, or ethical issues related to these data.

## Abbreviations

BCAS: Bilateral carotid artery stenosis
CC: Corpus callosum
IL-1*β*: Interleukin-1*β*
TNF-*α*: Tumor necrosis factor-*α*
TGF*β*1: Transforming growth factor-*β*1
IGF-1: Insulin-like growth factor-1
MBP: Myelin basic protein
LMP7: Low-molecular-mass peptide-7
UPS: Ubiquitin-proteasome system
CNS: Central nervous system
ELISA: Enzyme-linked immunosorbent assay
MCAO: Middle cerebral artery occlusion.

## Supplementary Materials

Supplementary Materials Supplemental Figure 1: effects of inhibition of LMP7 with PR957 on the neuroinflammation response in sham-operated groups. ELISA tested the levels of IL-1*β*, TNF-*α*, TGF*β*1, IGF-1, and IL-10 proteins in sham-operated groups treated with the vehicle or PR957. Data were expressed as mean ± standard deviation from three independent experiments. ^*∗*^*P* > 0.05, compared with the vehicle group; *n* = 10. Supplemental Figure 2: effects of inhibition of LMP7 with PR957 on the activation of TGF*β*/Smad signaling in sham-operated groups. Western blot confirmed the levels of TGF*β*1, Smad2/3, and phospho-Smad2/3 protein in sham-operated groups treated with the vehicle or PR957. Data were expressed as mean ± standard deviation from three independent experiments. ^*∗*^*P* > 0.05, compared with the vehicle group; *n* = 10.
