# Supplementary material for: Inhibition of the Immunoproteasome Subunit LMP7 Ameliorates Cerebral White Matter Demyelination Possibly via TGFβ/Smad Signaling
Source: Evid Based Complement Alternat Med. 2021 Oct 12;2021:6426225. doi: 10.1155/2021/6426225 (PMC8526201; doi:10.1155/2021/6426225)
Supplement: Supplementary Materials — Supplemental Figure 1: effects of inhibition of LMP7 with PR957 on the neuroinflammation response in sham-operated groups. ELISA tested the levels of IL-1β, TNF-α, TGFβ1, IGF-1, and IL-10 proteins in sham-operated groups treated with the vehicle or PR957. Data were expressed as mean ± standard deviation from three independent experiments. ∗P > 0.05, compared with the vehicle group; n = 10. Supplemental Figure 2: effects of inhibition of LMP7 with PR957 on the activation of TGFβ/Smad signaling in sham-operated groups. Western blot confirmed the levels of TGFβ1, Smad2/3, and phospho-Smad2/3 protein in sham-operated groups treated with the vehicle or PR957. Data were expressed as mean ± standard deviation from three independent experiments. ∗P > 0.05, compared with the vehicle group; n = 10. [file 6426225.f1.zip › Supplemental figure2.pptx]

## Slide 1
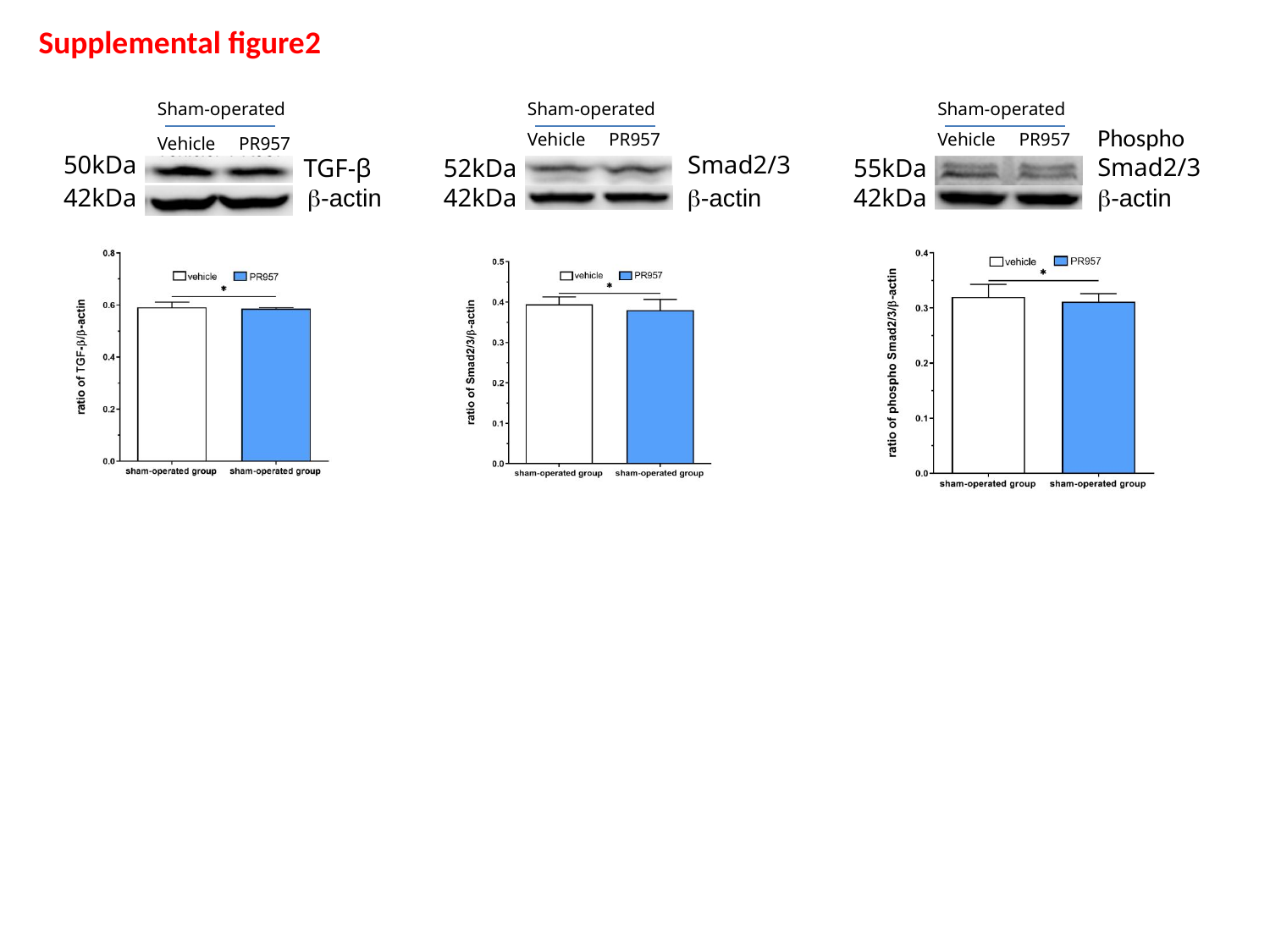

Supplemental figure2
Sham-operated
Sham-operated
Sham-operated
Phospho Smad2/3
Vehicle PR957
Vehicle PR957
Vehicle PR957
50kDa
Smad2/3
TGF-β
52kDa
55kDa
42kDa
b-actin
42kDa
b-actin
42kDa
b-actin
